# Dynamic procalcitonin-guided antibiotic stewardship among cancer patients in the UK

**DOI:** 10.1099/jmm.0.002145

**Published:** 2026-04-07

**Authors:** Dowan Kwon, Philip Williams, Helen Winter

**Affiliations:** 1 Bristol Royal Infirmary, University Hospitals Bristol and Weston NHS Foundation Trust, Bristol, UK; 2 Bristol Haematology and Oncology Centre, University Hospitals Bristol and Weston NHS Foundation Trust, Bristol, UK

**Keywords:** bacterial, cancer, infection, oncology, stewardship, procalcitonin

## Abstract

**Introduction.** Procalcitonin has been recognized as a tool for effective antibiotic stewardship to reduce unnecessary antibiotic use; however, its effectiveness remains unknown in the oncology setting, where infections are common and antibiotics are frequently prescribed.

**Gap statement.** The utility of procalcitonin for effective antibiotic stewardship in people with solid organ cancers is unknown.

**Aim.** To evaluate the role of procalcitonin dynamics in solid organ cancer patients with suspected bacterial infections in predicting clinical outcomes and guiding antibiotic therapy decisions.

**Methodology.** A single-centre evaluation was conducted at the Bristol Haematology and Oncology Centre, studying consecutive admissions of adult patients with solid organ cancer over a 3-month period. In a population in which serum procalcitonin levels were sporadically measured to guide antibiotic therapy, they were measured as standard care on admission and at 48 hours for patients admitted with a suspected bacterial infection. A threshold of 0.25 ng ml^−1^ was used to distinguish between low and high procalcitonin levels. Cases that had persistently low procalcitonin levels were retrospectively analysed for the potential identification of patients who could have had their antibiotic treatment ceased 48 hours into the antibiotic course.

**Results.** Seventy-seven cases with procalcitonin readings were recorded. Seventy (90.9%) cases received intravenous antibiotics during admission. Twenty-seven (35.1%) cases had persistently low procalcitonin, defined as <0.25 ng ml^−1^ on consecutive measurements, interpreted as unlikely to have bacterial infection as suggested from previous literature. No objective microbiological evidence of bacterial infection was observed in these cases. Retrospective clinician reviews of the 27 cases showed antibiotic therapy for 16 of the 27 cases could have been stopped 48 h into the admission, equivalent to a total reduction of up to 83/778 (10.7%) antibiotic days.

**Conclusion.** Procalcitonin could provide a helpful adjunct for clinicians to consider antibiotic stewardship and help reduce unnecessary antibiotic use in the oncology setting.

**Impact Statement**

Antimicrobial resistance is one of the largest global challenges that humanity faces. Widespread use of antibiotics contributes significantly to antibiotic resistance; therefore, better antibiotic stewardship to minimize unnecessary antibiotic use is imperative to minimize further antimicrobial resistance. Procalcitonin is a biomarker for bacterial infection that is more specific than conventional biomarkers, such as C-reactive protein and white blood cell count; however, there is very little literature on the use of procalcitonin for antibiotic stewardship in people with cancer.This study demonstrates that procalcitonin dynamics could be used to reduce unnecessary antibiotic therapy in the oncology setting by over 10%. Blinded prospective research is required to establish procalcitonin as a standard marker of bacterial infection to be used regularly in the oncology setting for adequate antibiotic stewardship.

## Data Summary

No data were generated or reused in the study.

The authors confirm all supporting data, code and protocols have been provided within the article or through supplementary data files.

## Introduction

Antimicrobial resistance (AMR) poses a significant threat to global health and the world economy. A global review and recommendations for tackling drug-resistant infections estimate that by 2050, the burden of deaths from AMR could reach 10 million deaths each year, with a cumulative cost to the global economy reaching 100 trillion USD [1]. These statistics equate to approximately one death every 3 s, and each person in the world is more than 10,000 USD worse off. This study explores the potential of procalcitonin (PCT) dynamics as a tool to aid antibiotic stewardship in patients with cancer.

PCT is a biomarker that has gained much attention in recent years to help discriminate between bacterial and viral diseases. PCT is produced from epithelial cells that encounter bacterial pathogens, and its expression is upregulated in the presence of bacterial pathogens and downregulated in patients with viral infections [2]. PCT level also decreases once the bacterial infection is controlled, providing useful information to clinicians regarding diagnosis and control [2]. It has a superior diagnostic accuracy for a wide range of bacterial infections including sepsis [2], bacterial pneumonia [3], bacterial co-infection with Coronavirus-19 virus [4], infected diabetic foot ulcer [5] and urinary tract infection [6], compared to more conventional and widely used biomarkers, such as white blood count (WBC) and C-reactive protein (CRP) [2].

An international experts’ consensus established by a Delphi process by Scheutz *et al*. proposed a PCT dynamics algorithm to promote improved antibiotic stewardship by measuring repeat PCT values within a 48-h window [7]. The consensus recommends using a cutoff figure of 0.25 ng ml^−1^ for patients with suspicions of bacterial infection outside the intensive care setting. This threshold of 0.25 ng ml^−1^ was utilized after it had been demonstrated on multiple settings, including emergency department and primary care, to successfully reduce antibiotic prescription rates without any increase in the risk for adverse outcome [8,9]. However, interpretation with caution is recommended on this consensus for patients with immunosuppression, as the majority of the studies that have led to this consensus have excluded those who are immunosuppressed. Further caution may be required as most literature around the use of the PCT threshold of 0.25 has focused on respiratory infections [7,8], with the association between PCT and other infection sources lacking.

PCT-based antibiotic stewardship in patients with cancer is still in its infancy and far from being widely utilized. Challenges and limitations exist in implementing PCT-guided strategies in real-world practice, emphasizing the need for further research and validation. This study evaluates the effectiveness of this algorithm, utilizing a cut-off figure of 0.25 ng ml^−1^, in guiding antibiotic stewardship in cancer patients.

Although PCT measurements were already used sporadically at the Bristol Haematology and Oncology Centre (BHOC), there has not been any routine standard of practice, including which patients had PCT measured to determine the likelihood of the presence of bacterial infection. Understanding PCT dynamics in patients with cancer would be essential to realize the benefits of PCT-based strategies in optimizing antibiotic use in this vulnerable population. Therefore, we introduced routine PCT measurements as standard care for those newly started on antibiotics to treat a bacterial infection. Our key aim was to evaluate those with consecutively low PCT values, divided across 48–72 h, to assess whether the PCT dynamics algorithm could be used to reduce unnecessary antibiotic use.

## Methods

A single-centre evaluation was conducted, which studied consecutive adult patients with cancer, admitted to the oncology ward at the BHOC for a suspected bacterial infection, between September 2021 and November 2021. Serum PCT measurement, a test that was already available to clinicians, was introduced as standard care for all new patient serum samples on admission and at ~48 h after admission. Patient demographics, cancer history and treatment summary, cancer treatment intent (curative or palliative) and the presence of indwelling intravenous catheters were also recorded. New antibiotic therapy was defined as at least a single dose of an antibacterial agent given in the hospital that the patient did not routinely receive, regardless of the route. Only patients with solid organ cancers were included in this study due to limited resources. Patients with haematological malignancies were excluded unless they were admitted with a concurrent solid organ cancer.

Cases were categorized based on PCT dynamics, using 0.25 ng ml^−1^ as a cut-off figure for both admission and 48 h serum PCT levels to define high and low PCT levels as suggested by the international expert consensus published by Schuetz *et al*. as outside the intensive care unit setting [7]. The cases were split into four groups: group 1, persistently low PCT; group 2, low PCT followed by high PCT; group 3, high PCT followed by low PCT; and group 4, persistently high PCT. Cases without two serial PCT measurements within 72 h of admission were excluded from the study. Twelve months following initial data collection, cases with persistently low serum PCT levels (group 1) were followed up using existing medical records, which included paper medical entries, electronic observations and discharge summaries. Investigations in biochemistry, haematology, microbiology and radiology were also recorded.

Group 1 cases, with persistently low serum PCT levels, were scrutinized by three clinicians within the study group (D.K., P.W. and H.W.) independently of each other, consisting of a resident medical doctor, a consultant medical microbiologist and a consultant medical oncologist, to assess the potential clinical utility of PCT dynamics for antibiotic stewardship in patients with cancer. The three clinicians were asked, ‘With hindsight, would you have stopped antibiotic treatment at 48–72 h if low serial PCT measurements were also available on top of all the other clinical information?’ with three solicited choices to choose from of ‘No’, ‘Possibly’ and ‘Probably’. The individual clinician’s decisions have been anonymized for publication.

Due to the retrospective nature of this clinical decision-making, we set ‘Probably’ as the most likely decision a clinician can make to demonstrate the likelihood of stopping the course of antibiotics and ‘No’ as the least likely. ‘Possibly’ was reserved for cases which were thought to be less clear-cut or potentially equivocal.

Antibiotic days saved were calculated by subtracting the number of antibiotic days patients received at the point of the second consecutive low PCT value being available from the total antibiotic course days that patients received.

### PCT measurement

Serum PCT was measured from venous blood samples taken in BD Vacutainer SST tubes from patients on admission and at ~48 h into the admission. Serum PCT measurements were performed by Elecsys BRAHMS Procalcitonin assay using the cobas e 602 module by Roche Diagnostics. The module was an electrochemiluminescence technology (patented). The measuring range was 0.02–100 ng ml^−1^, with values below and above this range reported as <0.02 ng ml^−1^ and >100 ng ml^−1^, respectively, with measurements reported to the nearest one decimal point.

### Ethics

This study was not considered as research, as defined by the UK policy Framework for Health and Social Care research, so it did not require Health Research Authority (HRA) or National Health Service ethics approval. This conclusion was reached after using the HRA decision tool [10] and after a discussion with the hospital’s research and development department. It was classed as a service improvement project. The research clinicians had access to patient data as part of their normal clinical care, and all data were stored and analysed on secure servers and conformed to local information governance requirements.

The study was not set out to test a clear and objective hypothesis but to evaluate the test that was already used to guide existing treatment practice at the BHOC.

Additionally, PCT was measured from serum samples that were already taken as the usual standard of care, with PCT measurements added to the samples. PCT measurement was often part of the usual service at the BHOC, and no additional samples were collected, nor any changes made to patient management.

## Results

**Fig. 1.**
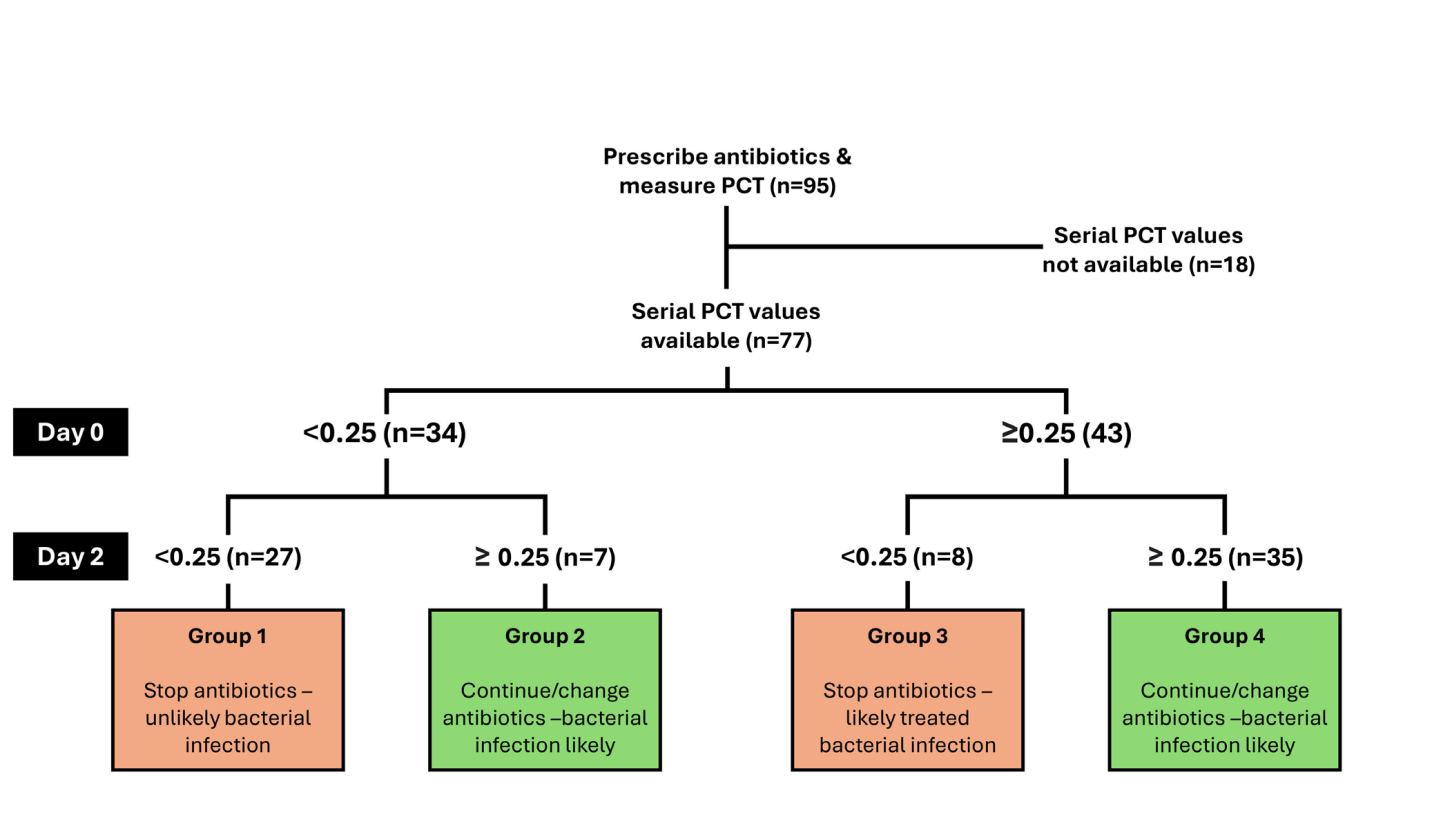
Flow diagram of total patient cases, adapted from the international consensus on using procalcitonin dynamics for antimicrobial stewardship by Schuetz *et al*. [7]. Prescribe antibiotics and measure (*n=95*, 95 hospital admissions from 86 cancer patients). Serial PCT values available (*n*=*77*, 39 female patients and 38 male patients). PCT, procalcitonin.

A total of 95 consecutive admissions were recorded over a 3-month data collection period from 86 inpatients. Six patients were admitted on more than one occasion with a separate episode of suspected bacterial infection within the data collection period. Eighteen cases were excluded as a result of not having consecutive PCT values within a pre-specified 72-h window following admission. Seventy-seven cases were split into the four groups: group 1 (persistently low PCT, *n*=27), group 2 (low PCT followed by high PCT, *n*=7), group 3 (high PCT followed by low PCT, *n*=8) and group 4 (persistently high PCT, *n*=35). Twenty-seven out of 77 (35.1%) cases were categorized in group 1, with persistently low PCT values, suggestive that they were unlikely to have had a bacterial infection in the first place. Eleven of the 27 (40.7%) cases were diagnosed as having had a respiratory tract infection on discharge. There were no cases of invasive bacterial infections, defined as positive blood culture growth, in the persistently low PCT value group. Fig. 1 shows this breakdown.

### Patient demographics and characteristics

A detailed breakdown of patient demographics and characteristics is shown in Table 1. There were 39 cases of female patients and 38 cases of male patients. The mean age was 58.5 years old. Breast (16, 20.8%), colorectal (10, 13.0%) and lung cancers (9, 11.7%) were the most prevalent cancers, with 48/77 (62.3%) having metastatic cancer at the time of admission and 55/77 (71.4%) receiving palliative treatment for the cancer. 40/77 (51.9%) were receiving chemotherapy alone, with 7/77 (9.1%) receiving no active cancer treatment for at least 6 months prior to admission. 22/77 (28.6%) had a peripherally inserted central catheter (PICC) at the time of admission.

**Table 1.** Patient demographics and characteristics. SD, standard deviation; PICC, peripherally inserted central catheter

Variable	Group 1 (persistently low PCT, *n*=27)	Group 2 (low PCT followed by high PCT, *n*=7)	Group 3 (high PCT followed by low PCT, *n*=8)	Group 4 (persistently high PCT, *n*=35)	Total (*n*=77)
**Sex**					
Female	15	5	3	16	39
Male	12	2	5	19	38
**Age (years), mean (sd), range**	62.3 (15.1), 21–80	56.3 (19.7), 28–75	51.4 (26.5), 17–79	57.6 (15.0), 24–83	58.5 (16.9), 17–83
**Primary cancer site**					
**Head and neck**	1	0	1	0	2
**Colorectal**	2	0	0	8	10
**Rest of the digestive tract**					
Oesophagus	1	1	0	1	3
Biliary tree	1	1	0	2	4
Pancreas	0	0	1	1	2
Anal	2	0	0	0	2
Small bowel	0	0	0	1	1
**Lung**	4	0	2	3	9
**Gynaecological**					
Fallopian tube	1	0	0	2	3
Ovarian	0	0	0	2	2
Vagina	1	0	0	0	1
Uterine	0	0	0	1	1
**Urinary tract**					
Prostate	4	0	0	3	7
Kidney	2	0	0	2	4
Bladder	0	0	0	1	1
**Breast**	5	4	2	5	16
**Mesothelial and soft tissue**					
Mesothelioma	1	0	0	0	1
Sarcoma	2	0	2	1	5
Germ cell	0	1	0	2	3
**Distant spread**					
Localized	12	5	4	7	28
Metastatic	15	2	4	28	48
**Treatment intent**					
Curative	6	5	5	6	22
Palliative	21	2	3	29	55
**Treatment type**					
Immunotherapy	3	0	1	2	6
Chemotherapy	13	5	5	17	40
Chemoradiotherapy	3	0	0	0	3
Combined immunotherapy and chemotherapy	1	0	1	0	2
No active treatment for >6 months (or better specified duration)	0	1	0	6	7
Targeted therapy	2	0	0	1	3
Radiotherapy	2	0	1	1	4
Combined immunotherapy and targeted therapy	1	0	0	0	1
Combined chemotherapy and targeted therapy	0	0	0	4	4
Hormone therapy	2	1	0	2	5
Combined hormone therapy and targeted therapy	0	0	0	2	2
**Indwelling vascular catheter**					
PICC	6	4	3	9	22
Central line	1	0	0	0	1
Port-a-cath	1	1	0	7	9
Nil	19	2	5	19	45

### Patient assessment and outcomes

25/77 (32.5%) cases had fever on admission, defined as a temperature measured as greater than 38.0. 74/77 (96.1%) had a quick Sepsis-Related Organ Failure Assessment (qSOFA) score of 0 to 1, indicating that patients were not at high risk for inpatient mortality with suspected infection. 10/78 (13.0%) were neutropenic on admission, defined as a neutrophil count of 0.5×10^9^ l^−1^ or less. Seventy (91.0%) were started on intravenous (IV) antibiotics, with the other seven on oral antibiotics. The mean length of stay was 6.4 days (sd=5.6; range 2–35) with a mean duration of antibiotic treatment of 10.2 days (sd=7.8; range 2–47), which included the antibiotic course that patients were discharged with.

Diagnosis and infection source were collected at three time points: on admission, 48 h into admission and at discharge. On initial assessment at admission, respiratory tract infections were the most common source (25/77, 32.5%), followed by gastrointestinal infections (15/77, 19.5%). Twenty-seven (35.1%) cases had an unspecified or unclear source of infection. Four (5.2%) cases had skin and soft tissue infection, exclusively all on their PICC insertion site. One (1.3%) patient was suspected of having infective endocarditis and another (1.3%) of a peri-anal abscess. There were no suspicions of a central nervous system infection upon initial assessment.

On discharge, the number of cases with skin and soft tissue infections, urinary tract infections and complex infections, including collections and abscesses, all rose. Seven (9.1%) cases were managed as having skin and soft tissue infections, once again, all from their respective PICC insertion site. Five (6.5%) cases were treated for urinary tract infection. In addition to the case treated for perianal abscess, a further two (2.6%) cases were treated as having a complex infection, which included an infected paraspinal collection and axillary abscess.

7/77 (9.1%) cases had significant positive blood culture results. Isolated pathogens were *Escherichia coli* (x2), *Staphylococcus epidermidis* (x2), methicillin-sensitive *Staphylococcus aureus*, *Klebsiella pneumoniae* and *Rhodotorula mucilaginosa*. Six of these had persistently high PCT levels, with the remaining one (containing *Staphylococcus epidermidis*) resulting in a dynamic increase of PCT of 0.2, which rose to 0.8. Interestingly, the patient with *R. mucilaginosa* had a PCT of 0.4 on admission and a subsequent result of 0.5 at 48 h into the admission, even though they had fungaemia without any objective evidence of bacterial infection. They were admitted with non-specific symptoms, including fevers, and were given 3 days of co-amoxiclav altogether to treat a bacterial infection empirically before starting an antifungal agent.

Additionally, there were two positive blood culture results that were considered likely contaminants by the local microbiology department following a clinical review (*Staphylococcus epidermidis* and *Staphylococcus haemolyticus*). Fifty-two (67.5%) cases did not develop any isolates in blood cultures, and 16 (20.8%) cases did not have any blood cultures taken (Tables2  3).

**Table 2.** Patient assessment and outcomes breakdown by PCT dynamics. PCT, procalcitonin; PTC, percutaneous transhepatic cholangiogram; SD, standard deviation; qSOFA, quick sequential organ failure assessment score

Variable	Group 1 (persistently low PCT, *n*=27)	Group 2 (low PCT followed by high PCT, *n*=7)	Group 3 (high PCT followed by low PCT, *n*=8)	Group 4 (persistently high PCT, *n*=35)	Total (*n*=77)
**Neutrophil count on admission**					
≤0.5	2	3	2	3	10
>0.5	25	4	6	32	67
**Fever on admission (>38.0)**	9	3	2	11	25
**qSOFA score**					
0-1	27	7	8	32	74
2-3	0	0	0	3	3
**New antibiotic therapy route on admission**					
Intravenous	23	7	8	29	70
Oral	4	0	0	3	7
**Length of stay (days), mean (sd), range**	6.1 (6.6), 2–35	3.9 (2.2), 2–8	7.8 (6.2), 3–19	7.1 (5.2), 2–21	6.4 (5.6), 2–35
**Duration of antibiotic treatment (days), mean (sd), range**	7.7 (2.8), 5–13	9.3 (2.9), 5–13	9.6 (4.6), 6–19	12.6 (10.7), 2–47	10.2 (7.8), 2–47
**Initial diagnosed infection source**					
Respiratory	13	0	5	7	25
Gastrointestinal	2	2	0	11	15
Skin and soft tissue (including catheter-associated infection)	4	0	0	0	4
Urinary	1	0	0	3	4
Complex infection (including abscess and collection)	1	0	0	0	1
Cardiovascular	1	0	0	0	1
Central nervous system	0	0	0	0	0
Unspecified/unclear	5	5	3	14	27
**Diagnosed the infection source on discharge**					
Respiratory	11	1	5	8	25
Gastrointestinal	2	1	0	12	15
Skin and soft tissue (including catheter-associated infection)	3	1	0	3	7
Urinary	2	0	1	2	5
Complex infection (including abscess and collection)	2	1	0	0	3
Cardiovascular	0	0	0	0	0
Central nervous system	0	0	0	0	0
Unspecified/unclear	7	3	2	10	22
**Blood cultures**					
Positive isolate (excluding likely contaminant, %)	0	1 (14.3)	0	6 (17.1)	7 (9.1)
Likely contaminant positive isolate (%)	0	0	0	2 (5.7)	2 (2.6)
No positive isolate (%)	18 (66.7)	5 (71.4)	6 (75.0)	23 (65.7)	52 (67.5)
Not taken (%)	9 (33.3)	1 (14.3)	2 (25.0)	4 (11.4)	16 (20.8)
**Urine cultures**					
Positive isolate (%)	3 (11.1)	0	2 (25.0)	3	8 (10.4)
No positive isolate (%)	9 (33.3)	3 (42.9)	0	7 (20)	19 (24.7)
Not taken (%)	15 (55.6)	4 (57.1)	6 (75.0)	25	50 (64.9)
**Respiratory secretion cultures**					
Positive isolate (%)	1 (3.7)	0	0	1 (3.7)	2 (2.6)
No positive isolate (%)	1 (3.7)	0	1 (12.5)	1 (2.9)	3 (3.9)
Not taken (%)	25 (92.6)	7 (100.0)	7 (87.5)	33	72 (93.5)
**Faeces PCR and cultures**					
Positive isolate (%)	0	1	0	3** (two are C.diff PCR only)	4
No positive isolate (%)	3	4	3	5	15
Not taken (%)	24	2	5	27	58
**Respiratory viral throat swab**					
Positive sample (%)	1	0	1	0	2
No positive sample (%)	9	3	2	11	25
Not taken (%)	17	4	5	24	50
**Coronavirus-19 rapid antigen test**					
Positive sample (%)	1	0	1	0	2
No positive sample (%)	25	7	7	35	74
Not taken (%)	1	0	0	0	1
Other microbiology tests					
Positive isolate (%)	1 (*Staphylococcus aureus* from left axilla abscess wound swab)			1 (*Pseudomonas aeruginosa*, *Escherichia coli*, *Streptococcus constellatus*, *Enterococcus faecium* from biliary PTC aspirate)	2
**28-day re-admission from presentation (%)**	4 (14.8)	3 (42.9)	0	11 (31.4)	18 (22.4)
**28-day mortality from presentation (%)**	1 (3.7)	1 (14.3)	1 (12.5)	3 (8.6)	6 (7.8)

**Table 3.** Pathogens identified

	Group 1 (persistently low PCT, *n*=27)	Group 2 (low PCT followed by high PCT, *n*=7)	Group 3 (high PCT followed by low PCT, *n*=8)	Group 4 (persistently high PCT, *n*=35)	Total (*n*=77)
**Pathogen isolated from blood culture**					
*Staphylococcus epidermidis*	0	1	0	2^a^	3
*Escherichia coli*	0	0	0	2	2
*Klebsiella pneumoniae*	0	0	0	1	1
*Rhodotorula mucilaginosa*	0	0	0	1	1
*Staphylococcus aureus*	0	0	0	1	1
*Staphylococcus haemolyticus*	0	0	0	1^b^	1
Total	0	1	0	8	9
**Pathogen isolated from urine culture**					
*Escherichia coli*	3	0	2	2	7
*Pseudomonas aeruginosa*	0	0	0	1	1
Total	3	0	2	3	8
**Pathogen isolated from respiratory secretion culture**					
*Haemophilus influenzae*	1	0	0	0	1
*Pseudomonas aeruginosa*	0	0	0	1	1
Total	1	0	0	1	2
Respiratory viral throat swab PCR*					
Coronavirus-19	0	0	1	0	1
Human metapneumovirus	1	0	0	0	1
Total	1	0	1	0	2
**Coronavirus-19 rapid antigen test**					
Coronavirus SARS-CoV-2	1	0	1	0	2
Total	1	0	1	0	2
**Pathogen isolated from faeces, PCR and culture**					
*Clostridium difficile*	0	1	0	3^c^	4
Total	0	1	0	3	4
**Pathogen isolated from other sites**					
*Staphylococcus aureus*		1^d^			1
*Pseudomonas aeruginosa*				1^e^	1
*Escherichia coli*				1^e^	1
*Streptococcus constellatus*				1^e^	1
*Enterococcus faecium*				1^e^	1
Total		1		4	5

aOne isolate was considered a contaminant. bThis isolate was considered a contaminant. cTwo isolates were PCR positive for *Clostridium difficile* toxic strain but toxin antigen negative.dThis isolate was from a wound swab of axilla abscess pus. eThese four isolates were all from the same sample, aspirated from a biliary percutaneous transhepatic cholangiography drain. *Respiratory PCR Multiplex Hologic by Cepheid was utilized, testing for influenza, adenovirus, human metapneumovirus, rhinovirus and parainfluenza.

### In-depth retrospective review of 27 cases with persistently low PCT values

**Fig. 2.**
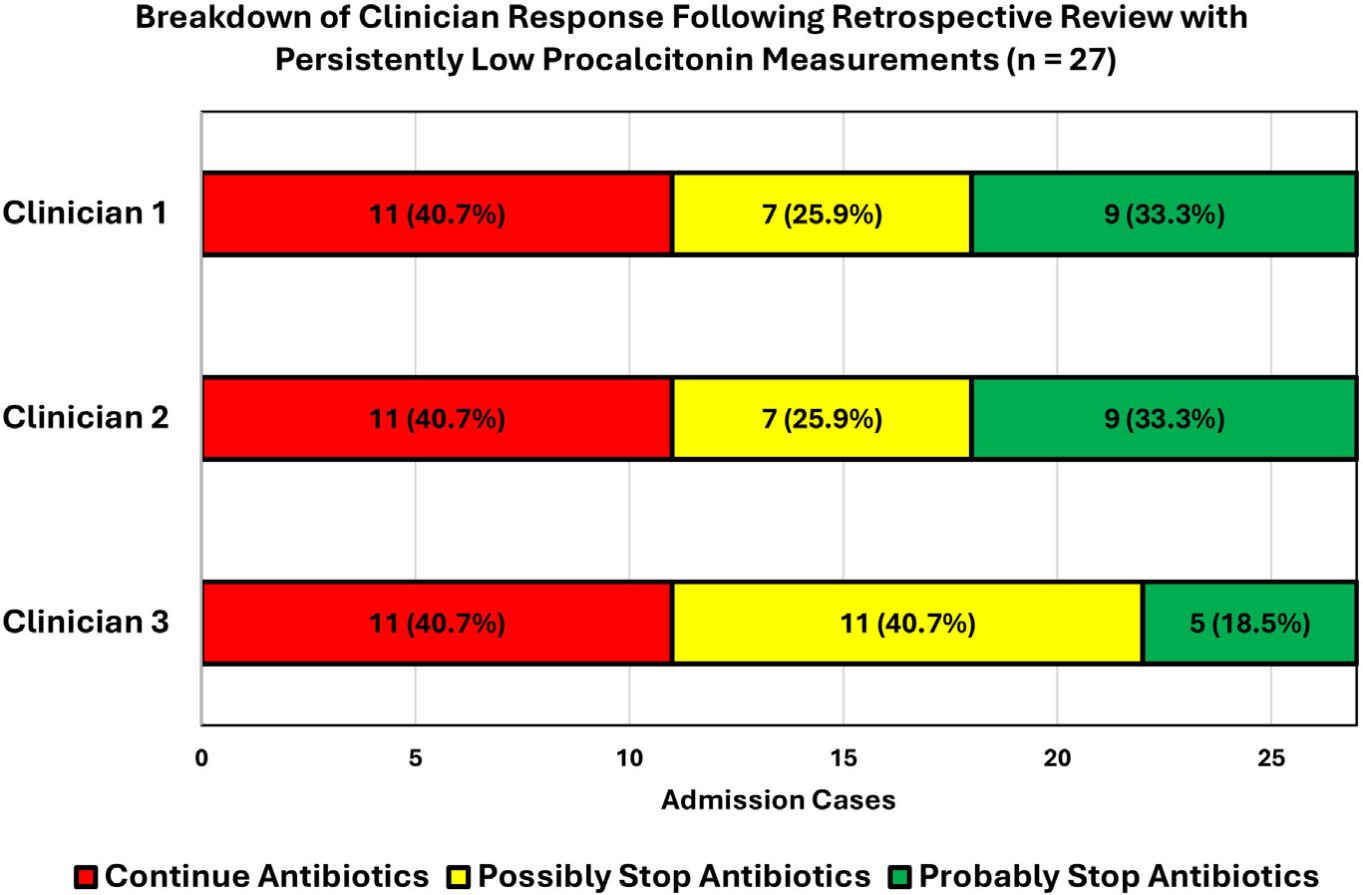
Retrospective clinical review outcomes for all group 1 (persistently low PCT values) cases. Clinicians selected between ‘No’, ‘Possibly’ and ‘Probably’ to the question ‘With hindsight, would you have stopped antibiotic treatment at 48–72 h if low serial PCT measurements were also available on top of all the other clinical information?’.

The clinical decisions on initial antibiotic course duration varied between the three independent retrospective reviews of the 27 cases. All three clinicians concluded that in the presence of all the clinical details, including the consecutive PCT values, they would have continued antibiotic treatment in 11 of the 27 cases, although not the same 11. They responded that they possibly or probably would have stopped antibiotic treatment at 48 h for the other 16 cases. The visual representation of these decisions is available from Fig. 2, with the full breakdown available from Table 4.

**Table 4.** Breakdown of retrospective clinical review outcomes for all group 1 (persistently low procalcitonin values) cases Clinicians were asked, ‘With hindsight, would you have stopped antibiotic treatment at 48–72 h if low serial PCT measurements were also available on top of all the other clinical information?’. Red, no; yellow, possibly; green, probably; blue, complete response concordance between three clinicians. CT, computed tomography; CRP, c-reactive protein; IV, intravenous; PICC, peripherally inserted central catheter; PO, Per os; PCT, procalcitonin.

Group 1** ** (*n*=27)	Brief description of the case at the time of the consecutive low procalcitonin level report	Antibiotic treatment on admission	Antibiotic treatment 48 h into admission	Clinician 1	Clinician 2	Clinician 3
**1**	Treated as cellulitis of the PICC insertion site	IV flucloxacillin	PO flucloxacillin	No	No	Probably
**2**	Fever on the background of chronic neutropenia; no infective source found	IV piperacillin With tazobactam	PO co-amoxiclav	Possibly	No	Possibly
**3**	Neutropenic after recent chemotherapy; new atrial flutter with cough and dyspnoea	IV co-amoxiclav	PO flucloxacillin+PO metronidazole	Probably	Probably	Possibly
**4**	New cough on immunotherapy; CT chest suggestive of dual pathology of pneumonitis and pneumonia	IV piperacillin with tazobactam	PO co-amoxiclav	Possibly	No	No
**5**	Fever and anal pain on the background of retroperitoneal cancer; CT showed anal tumour necrosis with no collection or abscess	IV co-amoxiclav	IV co-amoxiclav	Probably	Probably	No
**6**	Acute dyspnoea and orthopnoea; no lung pathology on CT; treated as infective exacerbation of chronic obstructive pulmonary disease	IV co-amoxiclav	IV co-amoxiclav+IV clarithromycin	Probably	Probably	Possibly
**7**	Fever and new pleural effusion; no growth on pleural aspirate; treated as pneumonia with malignant effusion	IV co-amoxiclav	IV co-amoxiclav+PO doxycycline	No	No	No
**8**	Fall at home with a change in behaviour; CRP in 300 s and new oxygen requirement; treated as sepsis of unknown source	IV co-amoxiclav	PO co-amoxiclav	No	Possibly	No
**9**	Fever with PICC insertion site cellulitis	PO amoxicillin	PO amoxicillin	No	No	No
**10**	Spontaneously ruptured peri-anal abscess; the surgical team recommended an antibiotic course, but not for any intervention	IV co-amoxiclav	IV co-amoxiclav	No	No	No
**11**	Known dysphagia and multiple previous aspiration episodes; treated as aspiration pneumonia	IV piperacillin with tazobactam	PO co-amoxiclav	Probably	Probably	Probably
**12**	Fever with recent CT scan showing possibly blocked biliary stent; no intervention planned as remained well	IV gentamicin	IV gentamicin	Possibly	Probably	Possibly
**13**	Urinary symptoms with fever and confusion; no growth on the urine sample	IV piperacillin with tazobactam	IV co-amoxiclav	No	Probably	No
**14**	Unilateral chest pain with fever on immunotherapy; CT suggested pneumonitis rather than pneumonia	IV vancomycin + IV piperacillin with tazobactam	PO flucloxacillin	Possibly	No	Possibly
**15**	Acute respiratory symptoms with CT suggestive of bilateral pneumonia	PO doxycycline	PO doxycycline	No	No	No
**16**	Acute respiratory symptoms soon after starting immunotherapy; CT scan inconclusive on infection vs. drug-induced pneumonitis	IV co-amoxiclav	IV co-amoxiclav	No	Possibly	Possibly
**17**	Fever with new murmur; echocardiogram largely normal and stable; no other infective source found and clinically well	PO amoxicillin	PO amoxicillin	Probably	Possibly	Possibly
**18**	Fever and sore throat with profound neutropenia	IV vancomycin	IV vancomycin	No	No	No
**19**	Fever with borderline neutropenia; no source found	IV piperacillin with tazobactam	PO co-amoxiclav	Possibly	Possibly	Possibly
**20**	Fever and myalgia after recent Coronavirus-19 vaccine; deranged liver function on immunotherapy	PO co-amoxiclav	PO co-amoxiclav	Probably	Probably	Probably
**21**	Small bowel obstruction found on CT; antibiotic started as temperature 38.0 on admission; no fevers recorded	IV co-amoxiclav	IV co-amoxiclav	Probably	Possibly	Probably
**22**	Acute dyspnoea; CT reported possible drug-induced pneumonitis; also treated for fluid overload	IV co-amoxiclav	IV co-amoxiclav	Possibly	Possibly	Possibly
**23**	Back pain with chronic respiratory symptoms; inconclusive CT findings with possible chronic changes only	IV co-amoxiclav	IV co-amoxiclav+PO doxycycline	Probably	Probably	Possibly
**24**	Fever, neutropenia and dyspnoea; CT showed pulmonary embolism but no pneumonia	IV ciprofloxacin + IV gentamicin + IV teicoplanin	IV co-trimoxazole	Possibly	Possibly	Possibly
**25**	New paraspinal collection on CT with fevers and CRP in 180 s; was awaiting drainage	IV co-amoxiclav	IV co-amoxiclav+PO doxycycline	No	No	No
**26**	Cellulitis at the site of recent PICC line removal; systemically well	IV piperacillin with tazobactam	IV piperacillin with tazobactam	No	No	No
**27**	Left-sided chest pain and arm weakness; CT showed progression of left lung cancer; no signs of infection	IV piperacillin with tazobactam	Nil	Probably	Probably	Probably

**Fig. 3.**
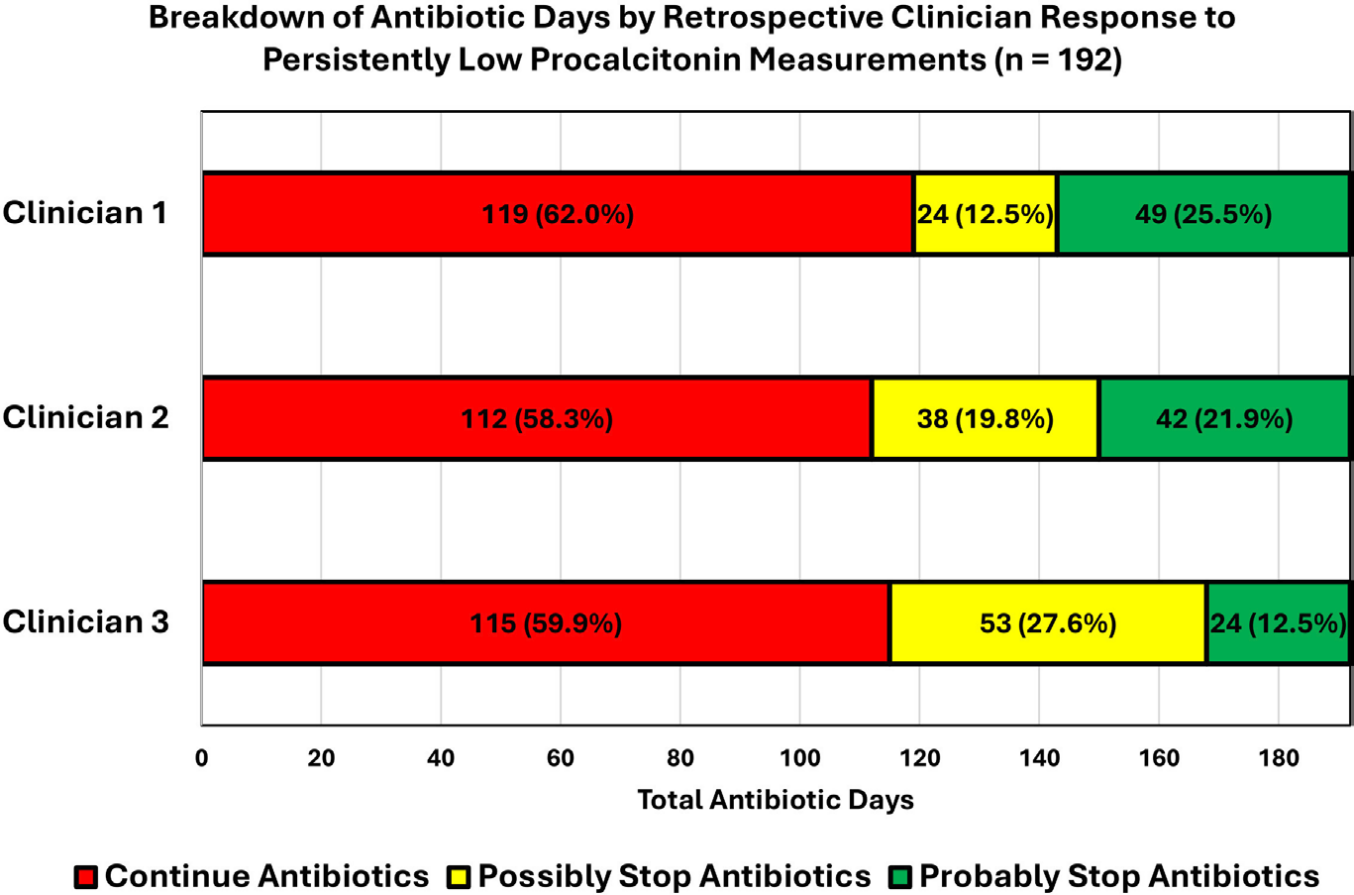
Total antibiotic days of group 1 (persistently low PCT values) cases broken down by retrospective clinician response. Clinicians selected between ‘No’, ‘Possibly’ and ‘Probably’ to the question ‘With hindsight, would you have stopped antibiotic treatment at 48–72 h if low serial PCT measurements were also available on top of all the other clinical information?’.

There was complete decision concordance between the 3 clinicians in 13 of the 27 cases (7× no, 3× possibly, 3× probably), with decision concordance between 2 clinicians for the other 14 cases. There was a total of 778 antibiotic days from 77 admissions over the 3-month period. The 27 cases of group 1 contributed to 192 antibiotic days. Going by individual clinician reviews, the number of antibiotic days saved ranged from 24 to 83 days, depending on the level of confidence that the clinician would have stopped antibiotics. To break this down, if we only count clinician 3’s ‘probably’ would have stopped the antibiotics category, 24 antibiotic days could have been saved initially, equivalent to 12.5% of antibiotic days from group 1 and 3.1% of all cases of 77 admissions. Alternatively, if we include clinician 1’s ‘possibly’ and ‘probably’ would have stopped antibiotics categories, 83 antibiotic days could have been saved, equivalent to 43.2% of antibiotic days from group 1 and 10.7% of all cases of 77 admissions. A visual representation of the potential antibiotic days saved are available from Fig. 3.

## Discussion

### Interpretation of results

To tackle AMR in the UK, the government had set out a target to reduce antibiotic use in humans by 15% over a 5-year period [11]. In this study, we have applied an existing international expert consensus guidance of PCT-guided antibiotic stewardship [7] to our cohort of patients with cancer. This is a particularly vulnerable population, with many of them significantly immunosuppressed either due to the cancer, the treatment or both. Getting antimicrobial treatment is crucial in this cohort, and we propose a potential reduction of up to 46.5% of antibiotic days for inpatients with consecutively low PCT values separated by 48 h.

This study also shows that PCT is not a robust tool to diagnose all bacterial infections in people with cancer. Even though there were 27 cases with PCT values lower than 0.25 ng ml^−1^ on 2 separate occasions, the retrospective reviews showed that each clinician would have continued antibiotics in at least 11 of the cases. These cases included a case of infected paraspinal collection, a case of perianal abscess and multiple cases of cellulitis of the PICC insertion site, for which the standard practice locally has been to treat with antibiotics. Radiology played a crucial role in antibiotic treatment in multiple cases, including a case of bilateral pneumonia and another of potential parapneumonic effusion.

Microbiological diagnosis is undoubtedly useful, but there are several causes of concern regarding microbiological reliance: (1) low yield of positive identifiable microbial growth, (2) sample contamination and (3) inability to differentiate between infection and colonization of non-sterile sites, such as urine and sputum [12,13]. Admittedly, a large part of our clinical decision-making still relies on empirical treatment, whereby we assess the patient’s clinical picture and response to antibiotics without any clear objective evidence of infection.

It is without a doubt that people with cancer remain clinically vulnerable to common community-acquired pathogens and opportunistic infections, especially those who are neutropenic. It remains to be seen whether the use of tools, such as PCT, could give clinicians enough confidence to stop unnecessary antibiotics, as the long-lasting culture of antibiotic overuse remains in many healthcare settings, meaning healthcare professionals will remain reluctant to stop antibiotics in people with cancer [14,16].

Different clinicians had different outcomes in retrospective reviews. For instance, clinician 3 was the most likely to continue the antibiotic course for the persistently low PCT group of cases. In theory, if all 77 cases of admissions were reviewed by clinician 3 with their antibiotic therapy reviewed with PCT dynamics, there could have been a reduction of at least 24–77 antibiotic days (3.1–9.9%). If all the cases were reviewed by clinician 1, these figures are higher at 49–83 antibiotic days (6.3–10.7%). As the retrospective clinical reviews were only held for the 27 cases with persistently low PCT, this figure could be an underestimate, as it is assumed that the clinicians would not have stopped the antibiotic course for any of the other patients who had at least one PCT value above 0.25 ng ml^−1^. It is possible that we could have considered stopping antibiotic therapy in patients in group 3 where the PCT levels dropped from above to below 0.25 ng ml^−1^. Retrospective clinical reviews by different clinicians were expected to lead to different outcomes as the clinicians were of different specialties and had differing clinical experience, too. Interpersonal variance in clinical decision-making is certainly not new [17], and different clinicians are likely to interpret investigations in different ways, too. This reflects the lack of a consistent and reliable method of assessment for a bacterial infection. However, we also felt that it was important to note that none of the 27 cases led to 3 strongly differing views from 3 clinicians. While there was not a complete concordance for all 27 cases, there was a decision concordance for all 27 cases between at least 2 of the 3 clinicians.

It should be noted that this is a very crude estimate, given that our study was of an evaluative nature and purely observational. Some of the clinical decisions to stop antibiotics may have been premature, leading to the patient deteriorating and antibiotics being restarted at a later date with a longer course prescribed. Promisingly, we showed that none of the cases with persistently low PCT had invasive bacterial infections, as shown by blood culture, or a high qSOFA score of 2 or more, indicating that none of the 27 cases were at high risk for in-hospital mortality with a suspected infection. Prospective research should be carried out to assess the impact of routinely utilizing PCT dynamics for inpatients with cancer suspected of having an acute bacterial infection.

### Procalcitonin cost-effectiveness

PCT has already been shown to be a cost-effective test in both intensive care and emergency department settings, leading to shorter hospital length of stay, reduction in medicines used and reduced antibiotic duration while leading to at least non-inferior care compared to standard practice [18,20]. Locally, PCT costs ~£12.00 per test in comparison to £4.38 for a serum full blood count test and £0.86 for a serum CRP. However, in the context of one-night admission in a general medical ward alone costing ~£400 without any added cost, such as medication, it is easy to see where the cost savings can be made. In our study, 23 out of 27 cases with persistently low PCT (group 1) received IV antibiotics on admission, and 14 remained on IV antibiotics at 48 h from admission. We predict that many of these patients would not have stayed as inpatients beyond the 48 h window had their antibiotic therapy been de-escalated, leading to a much shorter hospital stay.

### Potentially growing prevalence of cancer and immunotherapy efficacy with concurrent antibiotic use

Antibiotic stewardship in the context of progressive AMR has already been discussed and widely recognized internationally [1]. However, a less commonly discussed issue is the negative impact that antibiotic overuse can have, specifically on patients with cancer.

Firstly, cancer is already very common and perhaps becoming more prevalent. In England, both cancer incidence and survival have increased over the last 30 years, with mortality at 1 year, 5 years and 10 years all increasing at the same time [21]. In the UK alone, 4 million people are estimated to be living with cancer in 2030, compared to 1.2 million people and 2.5 million people in 1990 [22] and 2015, respectively [23]. With more people living with cancer, it is predictable that more people will be treated concurrently with systemic anti-cancer therapy and antibiotic therapy. This means that the impact of using antibiotics on the efficacy of cancer treatment should also be considered. With greater understanding of immuno-oncology, there has been a significant advance in the use of immunotherapy for people with non-small cell lung cancer, melanoma, renal cell carcinoma and many other cancers [24]. The concurrent use of antibiotic therapy with immune checkpoint inhibitors, some of the most used immunotherapy agents, is potentially detrimental, shortening cancer progression-free survival and overall survival of patients [25].

Although further validation studies are crucial, antibiotic exposure just before or after starting immune checkpoint inhibitor therapy seems to have a much larger effect in reducing the survival period compared to when used 90 days after initiation of immune checkpoint inhibitor therapy [26]. The MITRE trial is a currently ongoing multi-centre UK study which is primarily evaluating the gut microbiome as a biomarker to assess immune checkpoint inhibitor efficacy [27]. One of the eagerly awaited outcomes from the study is whether medicines like antibiotics could impact immunotherapy efficacy by disrupting the gut microbiome, which could impact how antibiotics are prescribed for patients on immunotherapy.

### Limitations

As previously mentioned, utilizing PCT in the oncology setting for antibiotic stewardship is relatively novel. Zhao *et al.* showed that PCT could be used to discriminate between tumour fever and infectious fever more accurately than current standard tests, such as CRP [28]. Chaftari *et al.* proposed a novel scoring system which utilized PCT, CRP and serum lactate to predict short-term mortality for cancer patients with suspected infection [29]. Morgan and Phillips showed that daily PCT could be used to discontinue antibiotics effectively for children who are febrile and neutropenic [30]. However, we found that in our cohort of 77 cases, the majority of the patients were non-neutropenic, non-febrile and not critically unwell with qSOFA scores of 0–1. RCTs have successfully shown that using PCT for patients in the general population with suspected bacterial infection can lead to reduced antibiotic exposure and result in non-inferior outcomes compared to standard practice. Gavazzi *et al.* showed that using PCT dynamics separated over 2 days with 107 participants in French geriatric units could successfully reduce antibiotic duration without compromising patient care [31], and Lhopitallier *et al.* showed that using PCT point-of-care testing in the primary care setting for acute cough can reduce antibiotic use significantly in a cohort of 469 people [32]. We propose that similar RCTs are required for patients with cancer.

Our study focused on inpatients only, with the consideration of using PCT dynamics to discontinue antibiotic therapy. However, we believe that looking into using PCT to switch IV antibiotics to the oral route may be of value too, potentially leading to shorter length of stay and decreased side effects from IV antibiotics and rates of hospital-acquired infections.

Only 27 cases with persistently low PCT (group 1) were evaluated in-depth due to limited resources, but in time, this would have been useful to expand to the entire 77 cases to see if retrospective clinical judgements in discontinuing antibiotics would have differed according to the PCT dynamic.

Performing a sub-group analysis for different tumour sites and types, and neutropenic cases was challenging due to the relatively small sample size of 77 cases and the great variance in tumour site, stage, systemic anti-cancer treatment type, progression and prognosis. In addition, Ichikawa *et al.* showed that PCT may be difficult to interpret in cases of small cell lung cancer (SCLC) as PCT is significantly raised in SCLC at baseline, with the mean value at 0.39 compared to 0.09 in non-small cell lung cancer [33]. Not only this, but the same study also showed that pre-treatment PCT can be used independently as a marker of survival, as it has a significant negative correlation.

Given the rich heterogeneity of cancer profiles, cancer clearly cannot be grouped as a single disease. Tumour site, stage, current treatment, progression and prognosis could all affect treatment, but clear guidance on antibiotic stewardship in the oncology setting is urgently required.

### Conclusions

This evaluation demonstrates that PCT dynamics may serve as a useful adjunct in antibiotic stewardship for oncology patients. Over one-third of inpatients with suspected bacterial infection had persistently low PCT levels, with retrospective review suggesting that antibiotics could have been safely discontinued in many of these cases. We estimate that this approach could reduce antibiotic days by up to 10.7% for oncology patients admitted with suspicions of an acute bacterial infection. This would represent an important step in addressing AMR as well as reducing the negative impact of unnecessary antibiotic use in a vulnerable population.

Although PCT should not replace clinical judgement, it provides greater specificity than conventional markers, such as CRP and WBC, and its dynamic trends supply additional objective evidence to support decision-making. Reassuringly, no invasive bacterial infections were observed in patients with persistently low PCT values.

Beyond stewardship, routine PCT testing may improve outcomes through shorter antibiotic courses, reduced hospital stays and avoidance of adverse effects from unnecessary antibiotics. This may be particularly relevant for patients receiving immunotherapy, where excessive antibiotic exposure could impair treatment efficacy.

Limitations include the study’s small, single-centre design and inter-clinician variability in retrospective decision-making. Future prospective multicentre trials are required to validate these findings and establish standardized practice. PCT has the potential to play an important role in improving infection management and antibiotic stewardship in the oncology setting.

## Abbreviations

AMR: antimicrobial resistance
BHOC: Bristol Haematology and Oncology Centre
CRP: C-reactive protein
CT: computed tomography
HRA: Health Research Authority
IV: intravenous
PCT: procalcitonin
PICC: peripherally inserted central catheter
PO: per os
PTC: percutaneous transhepatic cholangiogram
qSOFA: quick Sepsis-Related Organ Failure Assessment
SCLC: small cell lung cancer
